# Hypoglycemic Effects of *Sechium edule* (Chayote) in Older Adults: A Systematic Review and Meta-Analysis of Clinical and Preclinical Trials

**DOI:** 10.3390/foods14172937

**Published:** 2025-08-22

**Authors:** Taide Laurita Arista-Ugalde, Sebastián Delgado-Arroyo, Graciela Gavia-García, David Hernández-Álvarez, Itzen Aguiñiga-Sánchez, Edelmiro Santiago-Osorio, Juana Rosado-Pérez, Víctor Manuel Mendoza-Núñez

**Affiliations:** 1 Research Unit on Gerontology, FES Zaragoza, National Autonomous University of Mexico, Iztapalapa, Mexico City 09230, Mexico; tdlarista@comunidad.unam.mx (T.L.A.-U.); sebastda.21@gmail.com (S.D.-A.); hereda2912@gmail.com (D.H.-Á.); 2 Hematopoiesis and Leukemia Laboratory, Research Unit on Cell Differentiation and Cancer, FES Zaragoza, National Autonomous University of Mexico, Mexico City 09230, Mexico; itzen.aguiniga@zaragoza.unam.mx (I.A.-S.); edelmiro@unam.mx (E.S.-O.)

**Keywords:** hypoglycemic effect, metabolic syndrome, older adults, *Sechium edule*, chayote, type 2 diabetes mellitus

## Abstract

Type 2 diabetes mellitus (T2DM) and metabolic syndrome (MS) are chronic disorders characterized by hyperglycemia. *Sechium edule* (*S. edule*) has emerged as a complementary option due to its bioactive compounds. A systematic review of preclinical and clinical studies was carried out until 25 May 2025 in the databases PubMed, Scopus, Web of Science, SciELO, and TESIUNAM. The keywords were “diabetes mellitus”, “*Sechium edule*”, “Squash”, “Chayote”, “hypoglycemic effect”, and “Older adults”. A total of 110 articles were found; 11 met eligibility criteria (six clinical trials and five preclinical trials). Three clinical trials met the requirements for meta-analysis. The mean difference (MD) was calculated, and data were analyzed using RevMan 5.4 software. The meta-analysis showed a statistically significant decrease in serum glucose after three months (MD = −20.56, 95% CI −29.35 to −11.77, *p* < 0.0001) and six months after intervention (MD = −12.96, 95% CI = −21.90 to −4.02, *p* = 0.004). Likewise, there was a significant decrease in glycosylated hemoglobin (HbA1c) after three months (MD = −1.12, 95% CI = −1.45, −0.78, *p* < 0.0001) and after six months of intervention (MD = −0.92, 95% CI = −1.13, −0.25, *p* = 0.002). Our findings showed that *S. edule* intake has a statistically significant hypoglycemic effect in older adults with T2DM or MS by decreasing serum glucose and HbA1c levels. However, the magnitude of the decrease is clinically modest, so it cannot be a substitute for pharmacological treatment. For this reason, the intake of *S. edule* can only be considered as a complement to pharmacological treatment.

## 1. Introduction

Type 2 diabetes mellitus (T2DM) and metabolic syndrome (MS) represent major public health problems due to their high prevalence and strong association with serious complications, such as cardiovascular disease, kidney failure, and neuropathies. The older adult population, in particular, is at significant risk due to factors such as insulin resistance, pancreatic dysfunction, and the accumulation of comorbidities [1,2].

MS is a clinical entity that encompasses various metabolic alterations that predispose to the development of chronic diseases. MS is defined by the presence of at least three of the following factors: central obesity, dyslipidemia, hypertension, hyperglycemia, and low levels of HDL (high-density lipoprotein). This condition is closely associated with a significantly increased risk of cardiovascular and metabolic diseases. Insulin resistance plays a central role in its pathophysiology, contributing to endothelial dysfunction, oxidative stress, and chronic low-grade inflammation. In Mexico, the prevalence of MS has shown a sustained increase in recent years, reinforcing the need for effective intervention strategies for its management and prevention [2,3].

T2DM is a disease characterized by progressive alterations in insulin secretion and action, which generate chronic hyperglycemia. Its pathophysiology involves peripheral insulin resistance, pancreatic beta-cell dysfunction, and glucose metabolism dysregulation. This progressive deterioration is associated with an increased risk of micro- and macrovascular complications, which negatively impact patients’ quality of life. Furthermore, the coexistence of T2DM with MS increases the risk of adverse cardiovascular events and accelerates disease progression [4,5,6].

Allopathic treatment of T2DM is based on the use of hypoglycemic drugs, such as metformin, sulfonylureas, and DPP-4 inhibitors, among others. However, therapeutic adherence and adverse effects limit their effectiveness in many patients, which has motivated the search for natural therapeutic alternatives. In this context, nutraceuticals have gained importance as complementary options to improve glycemic control. Therefore, research into therapeutic alternatives of natural origin has gained relevance in recent years [7].

“Chayote”, whose scientific name is *Sechium edule* (*S. edule*), has been the subject of growing interest due to its potential hypoglycemic effects and its potential use as a therapeutic complement in the treatment of T2DM. This fruit, widely consumed in Mexico and other Latin American countries, has been shown to contain bioactive compounds with beneficial properties for glucose metabolism. Previous studies have identified the presence of flavonoids, polyphenols, and cucurbitacins in *S. edule*, which could play a key role in regulating glucose uptake in peripheral tissues and improving insulin sensitivity, among many other effects (Supplement S1). Furthermore, some studies have suggested that its regular consumption could contribute to the modulation of metabolic pathways involved in glycemic homeostasis, making it a promising option for inclusion in complementary therapeutic strategies for the management of T2DM (Supplement S2) [8,9,10,11,12].

The aim of this systematic review (SR) and meta-analysis (MA) is to present a synthesis of knowledge from preclinical and clinical trials on the hypoglycemic effect of *S. edules* in older adults with T2DM or MS.

## 2. Materials and Methods

### 2.1. Search Strategy

The SR was conducted according to the PRISMA-2020 guidelines (Preferred Reporting Items for Systematic Reviews and Meta-Analyses). The information search was conducted on scientific information search platforms such as PubMed, Web of Science, Scopus, LILACS, SciELO, and the gray literature (TESIUNAM). Using the acronym PICO (Population, Intervention, Comparator, Outcome), the following categories were established: P: humans and laboratory animals, I: consumption of *Sechium edule*, C: placebo or control, and O: glucose concentration and/or glycosylated hemoglobin. The protocol was registered in INPLASY (DOI:10.37766/inplasy2025.4.0025).

The search strategy was tailored to each of the search platforms. In the case of PubMed, English terms and Boolean operators and structured supplementary concepts were used in the strategy such as “Hypoglycemic effect” AND “*Sechium edule*”, “Diabetes mellitus” AND “hypoglycemic effect” AND [Squash OR “*Sechium edule”*] AND “older adults”, and [“*Sechium edule*” OR “squash”] AND “diabetes mellitus AND “laboratory animals”. In Web of Science, the strategies and Boolean operators “hypoglycemic effect” AND “*Sechium edule*”, “hypoglycemic effect” AND [“*Sechium edule*” OR Squash] AND “older adults” were used. For Scopus, only one search strategy was used, which was “hypoglycemic effect” AND [“*Sechium edule*” OR Squash] AND “older adults”. In the case of the Latin American platforms, SciELO and LILACS, the same search strategy was used, this time consisting of by Spanish words and Boolean operators such as “Hypoglycemic effect” AND “diabetes mellitus” AND [chayote OR “*Sechium edule*”]. Finally, in the case of the gray literature, the search strategy “Hypoglycemic effect” AND “*Sechium edule*” was used on the TESIUNAM platform. All records of the studies found during the search were entered into an Excel database to select those that were potentially included in the study.

### 2.2. Study Selection

Two reviewers (T.L.A.-U. and S.D.-A.) independently reviewed and assessed all titles and abstracts to identify studies via the inclusion criteria and excluded non-relevant studies. The reviewers were blinded to each other’s decisions. Discrepancies were discussed and resolved with another reviewer (J.R.-P.).

### 2.3. Inclusion Criteria

Inclusion criteria were as follows: (i) randomized clinical trials and pre-experimental studies in Spanish, English, and/or Portuguese; (ii) the use of *S. edule* as a treatment for diabetes or metabolic syndrome; (iii) placebo-controlled studies; (iv) evaluation of at least one of the following biochemical markers: serum glucose, HbA1c; and (v) the participation of older adults or laboratory animals regardless of gender, diagnosed with type 2 diabetes mellitus or MS.

### 2.4. Exclusion Criteria

Exclusion criteria were as follows: (i) studies that administered *S. edule* in combination with other compounds or medications; (ii) patients with gestational diabetes or prediabetics; (iii) observational studies; and (iv) randomized clinical trials that administered another species of *Sechium* spp.

### 2.5. Data Extraction

The following information was extracted from the selected full-text articles: surname and first name of the primary author and year of publication; study population for the intervention; frequency and dose used; mean, standard deviation, and sample size for the control and experimental group for each of the measurements for each marker; and the overall mean and standard deviation difference for each article. For MA, the number of participants in each intervention, the standard deviation, and the average obtained from the mean and standard deviation difference were extracted.

### 2.6. Risk of Bias Assessment

The risk of bias of the included articles was assessed using the RoB2 scale for randomized clinical studies, and the SYRCLE tool was used to assess the risk of bias of preclinical trials.

### 2.7. Statistical Analysis

To estimate the efficacy of *S. edule* treatment on biochemical markers of diabetes compared to placebo, an MA was performed for dichotomous variables using a random-effects model with the odds ratio (OR) as the measure of association.

Due to the high variability and heterogeneity in the preclinical or pre-experimental trials used for the SR, the decision was made to perform the meta-analysis only for randomized clinical trials. In these studies, the presentation or pharmaceutical form administered to participants was consistent across the five articles involved, as was the dose of 1500g per day. There was only variation in the timing of intervention administration and parameter measurement. Therefore, studies were stratified according to treatment duration into a group that measured the effects six months after the intervention and a subgroup of studies that measured the effects at three and six months after the intervention. This was performed for the two diabetes markers of interest studied (serum glucose and HbA1c). Therefore, independent MA were performed for each of them.

The SD of the global mean differences was determined using the following formula in the Microsoft Excel program:

$$\begin{array}{l} SDdifference=\\ \sqrt{{\left[(SD\;pretreatment\right)}^{2}+{\left(SD\;post\;treatment\right)}^{2}-(2\times R\times SD\;pretreatment\;\times \;SD\;post\;treatment)]} \end{array}$$

The Review Manager (RevMan) version 5.4 program was used to perform the MA. Pooled means and mean differences (MD) with 95% confidence intervals (CI 95%) were obtained; standard deviations were also calculated from the mean and standard error. To assess heterogeneity, I^2^ statistical analysis was performed, where a value ≤ 25% indicated no heterogeneity, 25–50% slight heterogeneity, 50–75% moderate heterogeneity, and >75% significant heterogeneity.

## 3. Results

**Figure 1:**
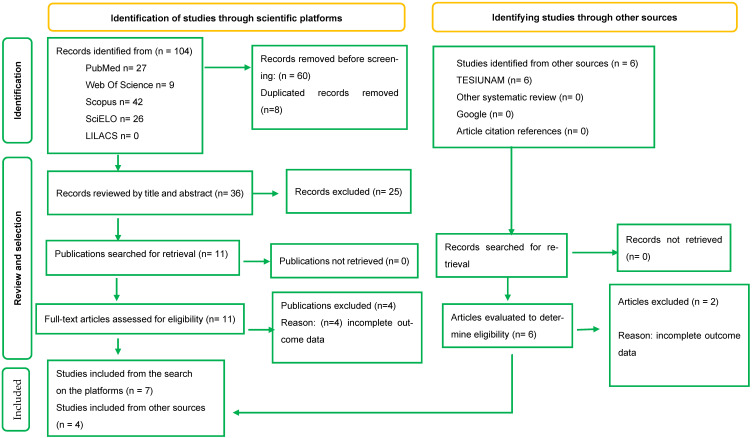
Process for selecting studies that met the eligibility criteria (PRISMA-2020).

With the established search strategies, 110 articles were found on the consulted platforms. After removing duplicates, the titles and abstracts of 42 articles were evaluated, and seventeen articles were selected for full-text review, of which eleven fully met the eligibility SR criteria, and only three clinical trials met the homogeneity criteria for quantitative analysis or MA (Figure 1).

### 3.1. Included Studies Characteristics

The sample size of the six articles included in the SR of the clinical trials was 282 older adults ranging in age from 60 to 71 years, all with diagnosed MS. The dose of *S. edule* extract consumed by patients in all studies was 500 mg three times per day, and the intervention time was from 1.5 months, 3 months, and 6 months (Table 1).

For the preclinical trials, the population consisted of 95 laboratory animals, primarily Wistar rats and *Mus musculus mice*. The heterogeneity of the studies included was high, as there was variation in the method used to induce diabetes in the laboratory animals and the type and dose of *S. edule* extract administered (Table 2).

### 3.2. Risk of Bias Analysis

**Figure 2:**
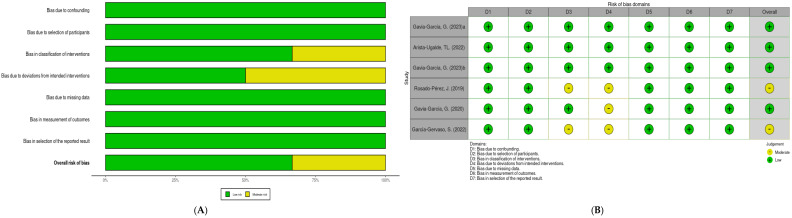
Clinical trial risk of bias: (**A**) summary risk of bias; (**B**) risk of bias per study [13,14,15,16,17,18].

Clinical Trials: A low risk of bias was identified in random sequence generation and allocation concealment in four of the six studies. However, two studies presented a moderate risk due to a lack of detail in the randomization methodology. Detection bias was low in most trials, but one study did not specify whether assessors were blinded to treatment. Reporting bias was low in all included studies (Figure 2).

**Table 1:** Effect of *Sechium edule* consumption on markers of hyperglycemia in clinical trials.

Author (Year)	Design	Population (n)	Dosage and Treatment	Treatment	Outcome																					
				Duration																						
Gavia-García et al. (2023) [13]	Clinical trial	48 elderly people with MS (PG *n* = 23, IG, *n* = 25),	IG: 1 capsule of 500 mg of dry powder of *S. edule* 3 times per day. PG: 500 mg placebo in 1 capsule 3 times per day.	6 months	Mean and SD pre- and post-intervention by study group																					
					Serum glucose (mg/dL)											%HbA1c										
					Placebo					*S. edule*						Placebo						*S. edule*				
					Baseline			6 months		Baseline				6 months		Baseline			6 months			Baseline		6 months		
					118 ± 67			123 ± 48		117 ± 73				108 ± 52		6.98 ± 1.6			6.94 ± 2			7.2 ± 1.2		6.28 ± 1.2		
					MD: −14 [CI 95%: −38.00, 10.00]											MD: −0.96 [CI 95%: −1.53, −0.39]										
Arista-Ugalde et al. (2022) [14]	Clinical trial	81 adults over 60 year with MS (PG *n* = 40, IG *n* = 41)	IG: 1 capsule of 500 mg of dry powder of *S. edule* 3 times per day. PG: 500 mg placebo in 1 capsule 3 times per day.	3 and 6 months	Mean and SD pre- and post-intervention by study group																					
					Serum glucose (mg/dL)											%HbA1c										
					Placebo					*S. edule*						Placebo						*S. edule*				
					Baseline	3 month			6 month	Baseline		3 month			6 month	Baseline	3 month			6 month		Baseline	3 month			6 month
					113 ± 43	123 ± 43			118 ± 40	113 ± 37		105 ± 29			103 ± 28	6.8 ± 1.9	6.5 ± 1.8			6.7 ± 1.9		6.3 ± 1.2	5.6 ± 1.2			5.9 ± 0.8
					MD (0–3 Months): −18 [CI 95% −28.82, −7.18]											MD (0–3 Months): −1.00 [CI 95% −1.43, −0.57]										
					MD (0–6 Months): −15 [CI 95% −25.65, −4.35]											MD (0–6 Months): −0.50 [CI 95% −0.93, −0.07]										
Gavia-García et al. (2023) [15]	Clinical trial	46 elderly people with MS (PG *n* = 20, IG, *n* = 26),	IG: 1 capsule of 500 mg of dry powder of *S. edule* 3 times per day. PG: 500 mg placebo in 1 capsule 3 times per day.	6 months	Mean and SD pre- and post-intervention by study group																					
					Serum glucose (mg/dL)											%HbA1c										
					Placebo					*S. edule*						Placebo						*S. edule*				
					Baseline			6 months		Baseline				6 months		Baseline			6 months			Baseline		6 months		
					137.3 ± 61.3			139.4 ± 69.1		140.7 ± 48.1				140 ± 57.3		N/D			N/D			N/D		N/D		
					MD: −2.80 [CI 95% −25.44, 19.84]																					
Rosado-Pérez et al. (2019) [16]	Pre-experimental exploratory	12 elderly people with MS.	500 mg of *Sechium edule* of dry poder in a capsule, 3 times per day,	1.5 months	Mean and SD values of baseline and post-treatment																					
					Serum glucose (mg/dL)											%HbA1c										
					*Sechium edule*											*Sechium edule*										
					Baseline						1.5 months					Baseline					1.5 months					
					107 ± 38						95 ± 5					5.9 ± 2.7					6 ± 2.7					
					MD: −12 [SD: 34.3]											MD: −0.1 [SD: 1.7]										
Gavia-García et al. (2020) [17]	Clinical trial	75 elderly people (PG n = 30, IG:, n = 45), with 67 ± 6 years of age with MS.	IG: 1 capsule of 500 mg of dry powder of *Sechium edule* 3 times per day. PG: 500 mg placebo in 1capsule 3 times per day.	3 months	Mean and SD values of baseline and post-treatment by study group																					
					Serum glucose (mg/dL)											%HbA1c										
					Placebo						*Sechium edule*					Placebo					*Sechium edule*					
					Baseline		3 months				Baseline		3 months			Baseline		3 months			Baseline				3 months	
					111 ± 57		121 ± 48				137 ± 72		125 ± 54			6.7 ± 1.9		6.3 ± 1.7			7.1 ± 1.9				6.2 ± 1.8	
					MD: −22 [C.I 95% −39.63, −4.37]											MD: −1.3 [C.I 95% −1.83, −0.77]										
García-Gervasio (2022) [18]	Clinical trial	20 elderly people PG n = 10, IG:, n = 10) 67–69 years old with MS.	IG: 1 capsule of 500 mg of dry powder of *Sechium edule* 3 times per day. PG, 500 mg placebo in 1capsule 3 times per day.	3 months	Mean and SD pre and post intervention by study group																					
					Serum glucose (mg/dL)											%HbA1c (mg/dL)										
					Placebo						*Sechium edule*					Placebo					*Sechium edule*					
					Baseline		3 months				Baseline		3 months			Baseline		3 months			Baseline				3 months	
					108 ± 47		123 ± 47				143 ± 56		123 ± 58			N/D		N/D			N/D				N/D	
					MD: −35 [C.I 95% −63.99, −6.01]																					

IG, intervention group; PG, placebo group.

**Table 2:** Effect of *Sechium edule* administration in preclinical trials.

Author (Year)	Design	Population (*n*)	Dosage and Treatment	Treatment Duration	Outcome									
Mohammad et al. (2024) [19]	Experimental	18 albino Wistar rats weighing 180–220g	Diabetic control (*n* = 6) with 60 mg/kg STZ. Two intervention groups with *Sechium edule* at 200 mg/kg (*n* = 6) and 400 mg/kg (*n* = 6).	28 days	Mean and SD pre- and post-intervention by study group									
					Serum glucose (mg/dL)									
					Control with T2DM				*S. edule*					
					60 mg/kg				200 mg/kg			400 mg/kg		
					Day 1		Day 28		Day 1		Day 28	Day 1		Day 28
					281.00 ± 2.56		369.35 ± 2.00		276.45 ± 3.01		165.88 ± 2.60	276.00 ± 1.95		152.00 ± 2.77

Aguiñiga-Sánchez et al. (2017) [20]	Experimental	20 CD-1 mice, 10 to 12 weeks old	A dose of 800 mg/kg of *S. edule* var. nigrum spinosum monitored for 7 days.	7 days	Mean and SD pre- and post-intervention by study group									
					Serum glucose (mg/dL)									
					Control					*S. edule*				
					Day 7					Day 7				
					115.72 ± 16.6					72.27 ± 3.8^2^				

Gómez-García (2013) [21]	Experimental	12 CD mice—1 female, 2 to 3 months old	A dose of 785 mg/kg of *S. edule* var. nigrum spinosum administered intraperitoneally every 48 h.	7 days	Mean and SD pre- and post-intervention by study group									
					Serum glucose (mg/dL)									
					Control					*S. edule*				
					Day 7					Day 7				
					105 ± 20					75 ± 16				

Montiel-García (2023) [22]	Experimental	10 Mus musculus L CD—1 mice, 8 to 10 weeks old.	Diabetic control with 175 mg/kg STZ. Four intervention groups with doses of 8 mg/kg, 50 mg/kg, 125 mg/kg, and 250 mg/kg of the hybrid *S. edule* H387-07.	30 days	Mean and SD pre- and post-intervention by study group									
					Serum glucose (mg/dL)									
					Control		*S. edule*							
					175 mg/kg		8 mg/kg		50 mg/kg		125 mg/kg		250 mg/kg	
					Day 1	Day 30	Day 1	Day 30	Day 1	Day 30	Day 1	Day 30	Day 1	Day 30
					433 ± 20	397 ± 18	451 ± 21	312 ± 19	372 ± 25	261 ± 20	440 ± 18	189 ± 12	420 ± 20	323 ± 18

Coria-Bárcenas (2017) [23]	Experimental	35 young male Wistar rats.	Two intervention groups in which 500 mg/kg of different *S. edule* extracts were tested every 48 h.	9 days	Mean and SD pre- and post-intervention by study group									
					Serum glucose (mg/dL)									
					Control		500 mg/kg of *S. edule* skin extract				500 mg/kg of *S. edule* pulp extract			
					Day 1		Day 1		Day 5	Day 7	Day 1		Day 5	Day 7
					103 ± 3.33		80 ± 5.25		82 ± 5.12	90 ± 13.3	75 ± 10		80 ± 12	81 ± 15

SD: standard deviation. STZ: streptozotocin.

**Figure 3:**
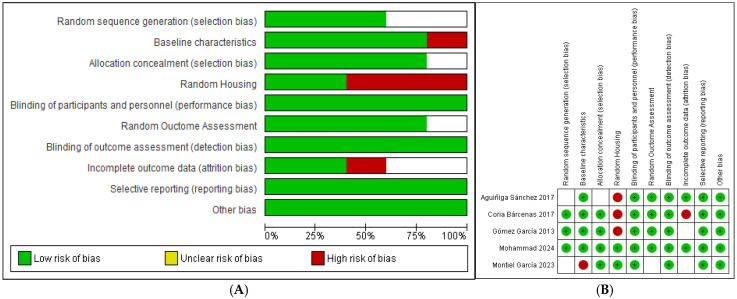
Preclinical trials risk of bias: (**A**) summary risk of bias; (**B**) risk of bias per study. Blank spaces represent moderate risks (SYRCLE tool) [19,20,21,22,23].

Preclinical Trials: The risk of bias in preclinical trials was assessed using SYRCLE’s RoB tool. A high risk of bias was identified in the random allocation of animals in three studies, as the randomization method was not speci-fied. Allocation concealment was not described in most studies, suggesting an unclear risk. The detection cycle was low in five studies, where investigators blinded the outcome assessment. However, attrition bias was high in two studies due to the exclusion of animals without a clear justification (Figure 3).

### 3.3. Serum Glucose

**Figure 4:**
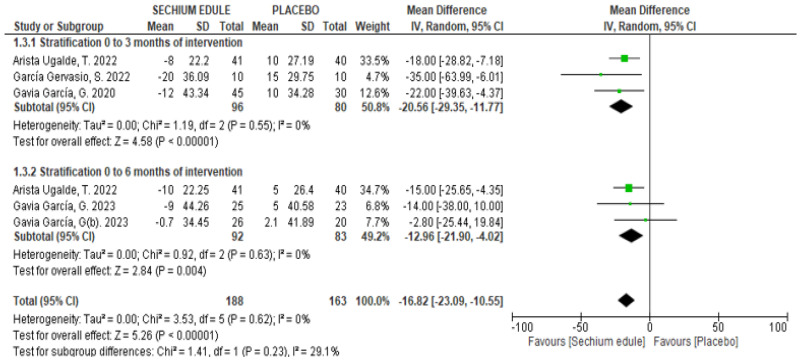
Effect of *S. edule* consumption on glucose levels in clinical trials stratified by intervention time [13,14,15,17,18].

*Clinical Trials*: Six clinical trials were included, which treated elderly patients ranging in age from 60 to 71 years. These studies observed a statistically significant decrease in serum glucose levels after treatment with *S. edule* capsules (Table 1). The MA of three studies showed MD = −20.56 mg/dL (CI_95%_: −29.35, −11.77, *p* < 0.00001) and MD = −12.96 mg/dL (CI_95%_: −21.90, −4.02, *p* = 0.004) after three and six months of treatment, respectively (Figure 4).

*Preclinical Trials:* Preclinical studies showed a decrease in glucose levels after administration of *S. edule* in the animal models (Table 2). However, due to the heterogeneity of the experimental designs and models used, a quantitative meta-analysis of the results was not possible.

### 3.4. Glycosylated Hemoglobin (HbA1c)

**Figure 5:**
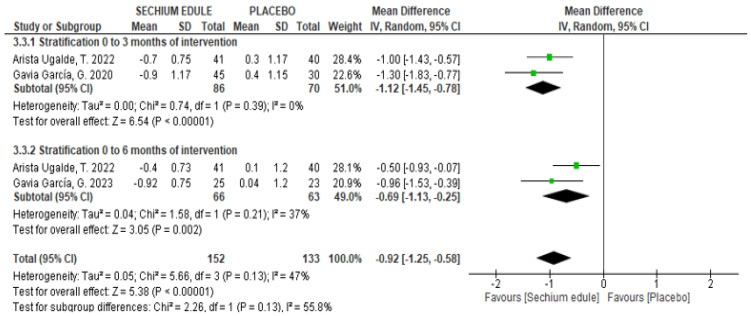
Effect of *S. edule* consumption on HbA1c levels in clinical trials stratified by intervention time [13,14,17].

*Clinical trials*: A significant reduction in the levels of HbA1c was observed after intake of *S. edule* capsules (Table 1). The MA showed MD = −1.12 (CI_95%_: −1.45, −0.78, *p* < 0.00001) and MD = −0.92 (CI_95%_: −1.13, −0.25, *p* = 0.002) after three and six months of treatment, respectively (Figure 5).

*Preclinical trials:* The studies did not show a statistically significant effect of *S. edule* consumption on this biomarker.

## 4. Discussion

Chronic hyperglycemia, characteristic of T2DM, has been identified as the disorder responsible for the development of complications in patients. It has also been linked to the development of other diseases such as MS and even cancer [24,25]. Treatment involves a multidisciplinary approach and has become a challenge for health-care professionals [26].

In this regard, the use of natural therapeutic alternatives has gained increased attention in recent years, particularly due to its popularity among the older population. *S. edule* is a fruit whose use, based on empirical knowledge, has been maintained over the years. Evidence of its effectiveness has been growing and is attributed to the combination of bioactive compounds this fruit possesses [20,27,28].

In this sense, in this SR, most studies agree on a significant decrease in serum glucose after administration of *S. edule*, suggesting a potential blood glucose-lowering effect. The SR, both from preclinical studies and clinical trials, provides a detailed overview of the existing evidence on this effect despite the diversity of the included studies, which vary in terms of methodological design, sample size, duration of treatment, and variables of interest.

Regarding the clinical trials, the results show a consistent trend toward a reduction in serum glucose and HbA1c, although with variability in the magnitude of the effect. Factors such as dosage, duration of treatment, and baseline patient characteristics may have influenced the observed heterogeneity. Despite this, the consistency in the direction of the effect suggests a potential clinical benefit.

The quantitative analysis result, through the MA of serum glucose, reinforces the findings reported in the SR. In this analysis, the overall effect estimate indicates a significant reduction in blood glucose levels after administration of *S. edule*, with a confidence interval suggesting a generalized downward trend. Although heterogeneity exists between studies, it does not compromise the validity of the pooled estimate, as the sensitivity analysis confirms that the results are consistent despite methodological differences. This finding supports the hypothesis that *S. edule* consumption could be an adjuvant in the treatment of glycemic control [29].

The MA of clinical trials of HbA1c levels provides additional evidence on the hypoglycemic effect of *S. edule* consumption. The reduction in HbA1c levels observed in the analyzed studies suggests an improvement in sustained glycemic control during the three and six months of treatment, which is relevant in the context of the control of T2DM. Unlike serum glucose, whose levels can fluctuate depending on dietary intake and other factors, glycated hemoglobin reflects metabolic control over prolonged periods. The results of the MA showed a significant decrease, with a favorable trend in most individual studies. It is important to mention that several studies have methodological limitations, such as small sample size and lack of blinding, which highlights the need for future research with greater methodological rigor [30,31].

As can be seen, the magnitude of the hypoglycemic effect is consistent, occurring at three months and continuing after six months of intervention. In this regard, regarding the slight decrease observed between the effect at three months and six months, we can assume that at three months, the placebo effect plus the pharmacological effect on the IG was observed. Since it has been reported that in human interventions, the placebo effect can last three months and disappear after six months, we assume this slight decrease in the effect is observed. Furthermore, the magnitude of the decrease in blood glucose and HbA1c concentration is consistent, thus ruling out a possible placebo effect [32,33].

The magnitude of the decrease is clinically modest, since a 1% decrease in HbA1c or approximately 20 mg/dL of glucose is not sufficient to improve the degree of control in diabetic patients; however, it may be a complementary option to pharmacological treatment [30,34].

It is important to highlight the small number of studies found and the high heterogeneity of the preclinical studies.

However, the observed heterogeneity suggests the need for further research to determine the duration of treatment and the specific mechanisms involved.

Regarding preclinical studies, although evidence supports the hypoglycemic effect of *S. edule* in animal models, the heterogeneity of experimental designs limits the possibility of drawing definitive conclusions. The lack of standardization in dosage, administration time, and blood glucose measurement methods hampers comparability across studies. Furthermore, the high risk of bias in the random assignment of animals and the exclusion of subjects without clear justification represent methodological challenges that should be addressed in future research.

Nevertheless, it is worth mentioning some proposed mechanisms of action to explain the hypoglycemic effect observed. It has been reported that phenolic acids, flavonoids, and cucurbitacin can contribute to these actions. Regarding flavonoids, it has been reported that quercetin has antidiabetic effects since it promotes insulin secretion, improves insulin resistance, and maintains glucose homeostasis [35]. It has been shown to do so by various mechanisms, for example, promotion of insulin expression and glucose-stimulated insulin secretion in the INS1-E (insulin-secreting rat insulinoma) cells; in addition, it promotes the activation of ERK1 and ERK2, resulting in a significant increase in insulin secretion from pancreatic β1 cells. It has also been shown that it activates the intracellular Ca^2+^ signaling pathway [36,37,38].

Regarding rutin, the suggested mechanisms for its hypoglycemic effect comprise blockage of tissue gluconeogenesis, a reduction in carbohydrate assimilation from the small intestine, an improvement in tissue glucose uptake, stimulation of insulin secretion from beta cells, and protection of Langerhans islets. Rutin also reduces the generation of sorbitol, reactive oxygen species, advanced glycation end-product precursors, and inflammatory interleukins [39,40].

Phlorizin’s fundamental pharmacological action is to produce renal glycosuria and avoid intestinal glucose absorption by suppressing sodium–glucose symporters in the proximal renal tubule and mucosa of the small intestine [41].

Regarding cucurbitacins, it has been shown that cucurbitacin B could considerably decrease blood glucose in a diabetic mouse model. Its effect was principally realized by regulating the intestinal level of AMPK, stimulating the secretion of plasmatic glucagon-like peptide-1 and insulin, and then modifying mice’s appetite [42].

On the other hand, gallic acid (GA) has been observed to improve insulin sensitivity and glucose homeostasis in adipocytes. The proposed mechanism is through the activation of PPAR-γ and the promotion of GLUT4 translocation. Furthermore, GA could improve insulin sensitivity by regulating the Akt and AMPK signaling pathways, revealing the dual activation of Akt and AMPK. Likewise, the hypoglycemic effects of GA could be mediated by the regulation of TNF-α and adipocytokine expression. Furthermore, GA inhibits caspase-9-related cell apoptosis, thus improving cellular function [43,44].

Among the most important limitations, we can highlight the limited number of clinical and preclinical studies found, in addition to the high heterogeneity of the preclinical studies. No studies were found comparing *S. edule* with other foods or supplements with hypoglycemic effects. However, the hypoglycemic effects of *S. edule* make it a complementary option for the treatment of T2DM and MS.

### 4.1. Practical Implications

The results of the SR and MA showed a statistically significant reduction in glucose and HbA1c levels due to *S. edule* intake in preclinical studies and in older adults with T2DM or MS; however, the magnitude of the reduction is clinically modest. Therefore, *S. edule* consumption should only be included as a complement to pharmacological treatment, although it could be a good preventive option in patients with risk factors for T2DM and/or prediabetic subjects.

### 4.2. Research Implications

It would be advisable to compare the hypoglycemic effect of *S. edule* with other food options or nutritional supplements through SR with network MA. On the other hand, considering the modest clinical effect of *S. edule* intake on glucose levels for T2DM control, it would be advisable to conduct clinical trials that include pharmacological treatments in addition to *S. edule* intake, for example, metformin combined with *S. edule*.

## 5. Conclusions

The findings of this SR and MA showed that *S. edule* intake has a statistically significant hypoglycemic effect in older adults with T2DM or MS, reducing serum glucose and HbA1c levels. However, the magnitude of the decrease in these clinical markers is clinically modest enough to consider them as a substitute for pharmacological treatment for the control of T2DM. Therefore, it is advisable to carry out further clinical studies considering the combination of foods with hypoglycemic effect and/or combining pharmacological treatments with *S. edule.*

## Data Availability

The original contributions presented in this study are included in the article. Further inquiries can be directed to the corresponding authors.

## Supplementary Materials

The following supporting information can be downloaded at: https://www.mdpi.com/article/10.3390/foods14172937/s1, Supplement S1. Content of bioactive compounds present in 500 mg of dried fruit of *Sechium edule*, *Nigrum spinosum* varietal group (14); Supplement S2. *Sechium edule* bioactives and their hypoglycemic effects.

## Abbreviations

The following abbreviations are used in this manuscript:

CI	Confidence interval
DPP-4	Dipeptidyl peptudase-4
EG	Experimental group
GA	Gallic acid
HDL	High-density lipoprotein
INS1-E	Insulin-secreting rat insulinoma
MA	Meta-analyses
MD	Mean difference
MS	Metabolic syndrome
PRISMA	Preferred Reporting Items for Systematic Reviews and Meta-Analyses
*S. edule*	*Sechium edule*
SD	Standard deviation
SR	Systematic review
STZ	Streptozotocin
T2DM	Type 2 diabetes mellitus
