# Supplementary material for: Hypoglycemic Effects of Sechium edule (Chayote) in Older Adults: A Systematic Review and Meta-Analysis of Clinical and Preclinical Trials
Source: Foods. 2025 Aug 22;14(17):2937. doi: 10.3390/foods14172937 (PMC12428100; doi:10.3390/foods14172937)
Supplement: Supplementary file 1 [file foods-14-02937-s001.zip › foods-3815691-supplementary.pdf]

Supplement S1. Content of bioactive compounds present in 500 mg of dried fruit of *Sechium edule, nigrum spinosum* varietal group (14)

**Metabolite name**	**Concentratión (μg)**	**Estandar deviatión**
**Flavonoids**		
Rutin	45.49	12.44
Quercetin	1.30	0.176
Apigenin	0.014	0.007
Miricetin	2.38	0.129
Florizin	14.26	2.28
Naringenin	48.85	7.046
**Phenolic acids**		
Syringic acid	8.7	0.034
Protocatechuic acid	3.3	1.56
Caffeic acid	9.27	0.066
P-hydroxybenzic acid	0.115	0.019
Gallic acid	38.83	1.37
P-coumaric acid	1.69	0.23
Clorogénico acid	1.39	0.30
Ferulic acid	7.03	0.37
**Cucurbitacins**		
Cucurbitacin D	6.11	0.63
Cucurbitacin B	89.94	18.15
Cucurbitacin E	154.8	3.3
Cucurbitacin I	0.71	0.26

Independent duplicate analysis by High Performance Liquid Chromatography (HPLC).

Supplement S2. *Sechium edule* bioactives and their hypoglycemic effects

		Actions	Suggested Mechanisms
	Quercetin 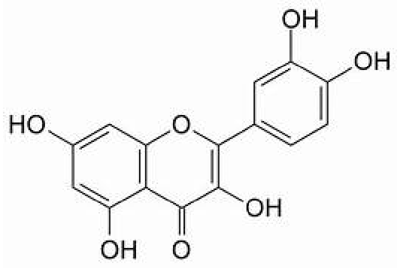	Promotes insulin secretion	Activating extracellular signal-regulated kinase 1/2 (ERK1/2) activating the intracellular Ca2+ signaling pathway [36].
		Improves insulin resistance	Upregulating the phosphorylation of insulin receptors (InsRs) upregulating the phosphorylation of protein kinase (PK) [37].
		Maintains glucose homeostasis	Induced AMPK activation (downregulating key glycogenic isoenzymes) stimulating the translocation of the glucose transporter type 4 (GLUT4) promote glucose processing by sirtuin 1 (SIRT1) [38].
Flavonoids	Rutin 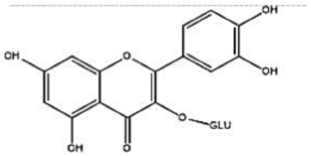	Diminishes hyperglycemia	Decrease carbohydrate absorption from the small intestine Inhibition of tissue gluconeogenesis. Increase in tissue glucose uptake. Stimulation of insulin secretion from beta cells, protection of Langerhans islet against degeneration [39-40].
	Phlorizin	Diminishes hyperglycemia .	Causes renal glycosuria by inhibiting the sodium–glucose symporters in the proximal renal tubule.

	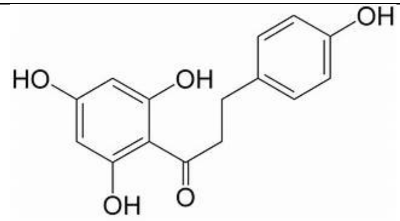		Avoid intestinal glucose absorption by regulating the mucosa of the small intestine [41].
Cucurbitacins	Cucurbitacin B 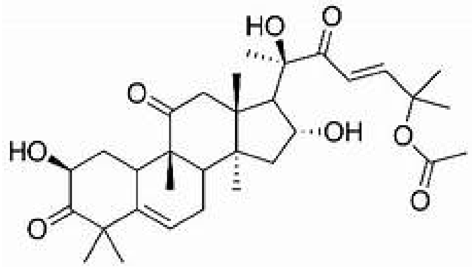	Reduce the blood glucose level	Regulating the intestinal level of AMPK, inducing the release of plasma glucagon-like peptide-1 and insulin [42].
Phenolic acid	Gallic acid 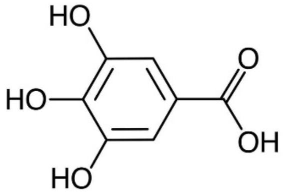	Facilitates insulin sensitivity and glucose homeostasis. .	Activation of PPAR- and C/EBPs. Promotion of GLUT4 translocation. Regulates Akt and AMPK signaling pathways [43-44].
